# Clinicians’ perspectives on quality: do they match accreditation standards?

**DOI:** 10.1186/s12960-021-00616-w

**Published:** 2021-06-19

**Authors:** Nesibe Akdemir, Romana Malik, Theanne Walters, Stanley Hamstra, Fedde Scheele

**Affiliations:** 1 School of Medical Sciences, VU Medical Center, Amsterdam, The Netherlands; 2 Department of Medical Education, OLVG Hospital, Amsterdam, The Netherlands; 3 Athena Institute for Transdisciplinary Research, Vrije Universiteit, Amsterdam, The Netherlands; 4 Australian Medical Council, Canberra, Australia; 5 Milestones Research and Evaluation, Accreditation Council for Graduate Medical Education, Chicago, IL United States of America; 6 Faculty of Education, University of Ottawa, Ottawa, ON Canada; 7 Department of Medical Education, Feinberg School of Medicine, Northwestern University, Chicago, IL United States of America

**Keywords:** Accreditation, Regulation, Quality improvement, Quality of training, Medical education

## Abstract

**Background:**

Quality of training is determined through programs’ compliance with accreditation standards, often set for a number of years. However, perspectives on quality of training within these standards may differ from the clinicians’ perspectives on quality of training. Knowledge on how standards relate to clinicians’ perspectives on quality of training is currently lacking yet is expected to lead to improved accreditation design.

**Methods:**

This qualitative study design was based on a case-study research approach. We analyzed accreditation standards and conducted 29 interviews with accreditors, clinical supervisors and trainees across Australia and the Netherlands about the quality and accreditation of specialist medical training programs. The perspectives were coded and either if applicable compared to national accreditation standards of both jurisdictions, or thematized to the way stakeholders encounter accreditation standards in practice.

**Results:**

There were two evident matches and four mismatches between the perspectives of clinicians and the accreditation standards. The matches are: (1) accreditation is necessary (2) trainees are the best source for quality measures. The mismatches are: (3) fundamental training aspects that accreditation standards do not capture: the balance between training and service provision, and trainee empowerment (4) using standards lack dynamism and (5) quality improvement; driven by standards or intrinsic motivation of healthcare professionals.

**Conclusion:**

In our Australian and Dutch health education cases accreditation is an accepted phenomenon which may be improved by trainee empowerment, a dynamic updating process of standards and by flexibility in its use.

## Background

Clinical supervisors have a key role in training future medical specialists. However, while valuable, their individual influence on the policies and criteria for accreditation of their training programs is usually limited, as is the influence of trainees [1–3]. Several studies have shown that clinical supervisors and trainees acknowledge the importance of standards in medical education [4–6], yet accreditation processes may collect data and information that does not always reflect the clinicians’ views on quality of training [7].

The system of accreditation of medical specialist training answers to the public and the medical society for the quality of the specialists produced [8]. Accreditation authorities set and monitor standards that oversee the training of future medical specialists [9]. In the context of this research accreditation is defined as a mandatory process by which an accreditation authority reviews the training program using a set of standards with a quality management approach [10]. The purpose of quality management is usually assessing, assuring and enhancing quality. It is a concept which originates in the manufacturing industry [11] and was defined as all activities designed to achieve and sustain high-quality output [12, 13]. These days, quality management of a product or service consists of three major approaches which could be used independently: quality assurance, quality improvement, and quality control [14]. In quality assurance compliance assessing (minimum) standards is ensured, whereas in quality improvement there is encouragement to move beyond the standards. Both above-mentioned quality approaches focus on the process of quality management, while quality control is focused on the output of the process. If needed standards should help accreditation authorities to address quality concerns. Ideally, following the standards should assure educational quality, promote (continuous) quality improvement and measure quality output and lead to high-quality medical specialist training.

Clinical supervisors and trainees have their own perspectives on what high-quality medical specialist training entails [15–17]. At the same time, external accreditation standards, which are usually the composite of the views of several societal and professional stakeholders, shape accreditation authorities’ views and assessment of the quality of training. Although accreditation standards often remain unchanged for a number of years, clinical training units are continuously subject to change under the influence of rapid developments in health care and medical education; clinical practice and societal developments have a more dynamic discourse than accreditation. This raises the question whether there is a gap between clinicians’ perspectives and the fixed perspectives behind the accreditation standards that clinicians follow.

The pilot study of Klessig et al. already showed that there might be a discrepancy between the existing accreditation requirements on the one hand and the quality indicators mentioned by clinical supervisors and trainees on the other hand [17]. Although this study indicates a potential discordance between these perspectives on quality of the training, research to date has not yet determined in which aspects the perspectives of clinicians differ from the accreditation standards.

Alignment of these perspectives may contribute to a more evidence informed accreditation design for continuous quality improvement of training [9, 18]. Therefore, this qualitative study aims to determine important quality aspects regarding postgraduate medical training according to clinicians’ perspectives, how these aspects relate to accreditation standards, and how clinicians encounter accreditation standards in practice.

## Methods

### Study design

This qualitative study was based on a case-study research approach, in which a phenomenon is examined within its real-life context [19]. This enabled a holistic view of specific, contextualized cases to understand complex situations and deepen knowledge [20]. We explored the variation and underlying issues in different stakeholders’ experiences of the quality and accreditation of medical specialist training in Australia, a new system established in 2002, and the Netherlands, a well-established system since 1961.

We considered the perspectives of clinical supervisors, trainees and accreditors on the quality and accreditation of medical specialist training by conducting in-depth interviews. In addition, accreditation standards from both jurisdictions were collected for document analysis.

### Participants and procedure

The study included two groups of stakeholders. The group of clinicians consisted of clinical supervisors and trainees involved in daily clinical practice. We included a second group to deepen our understanding of accreditation standards, consisting of accreditors that contributed to the development of standards.

The criteria for participation were:

- Clinical supervisors and trainees from accredited medical specialist training programs in Australia and the Netherlands.
- Members of the Specialist Education Accreditation Committee (SEAC) from the Australian Medical Council (AMC, Australia), and accreditors from the Dutch Legislative College for Accreditation of Postgraduate Medical Education and Specialist Registration Committee (RGS, CGS, the Netherlands).

The participants in category (a) were not accreditors or surveyors in the accreditation of medical specialist training programs mentioned in category (b).

### Recruitment of participants

Australian supervisors, trainees and accreditors were sourced through AMC affiliates. Dutch supervisors and trainees from different departments and hospitals were invited through the secretary of the hospital department, Dutch accreditors through the Secretary of the Dutch Board for Accreditation of Postgraduate Medical Education and Specialist Registration Committee (CGS, RGS the Netherlands).

### Data collection

A total of 44 stakeholders were invited of whom 29 participated (Table 1). Semi-structured face-to-face or telephone interviews lasted 45–60 min. Interviews were conducted until data reached theoretical sufficiency [21]. Participants were asked the following: (1) what are the most important aspects of a high-quality training program? (2) How would you monitor these aspects if you were the accreditation authority?

**Table 1:** Participants

Stakeholders	AU	NL
Invited	26	18
Participated	16	13
Clinical supervisors	5	4
Trainees	5	5
Accreditors	6	4

The Australian and Dutch national standards were collected, respectively, the ‘Standards for Assessment and Accreditation of Specialist Medical Programs and Professional Development Programs by the Australian Medical Council 2015’[22] and ‘Standards for Postgraduate Medical Education Accreditation by the Legislative College 2016’ [23].

### Data analysis

Data were analyzed in three phases. Firstly, the audiotaped interviews were transcribed verbatim. Anonymized transcripts were analyzed thematically and line-by-line using open coding to explore (1) all quality aspects of training (2) stakeholders’ experiences of using accreditation standards in practice. This resulted in code scheme 1 and 2, respectively.

In the second phase, accreditation standards of both jurisdictions were analyzed with open coding and were combined with the accreditor’s transcripts of code scheme 1, which resulted in code scheme 3. MaxQDA version 2018 was used for coding.

RM coded the data as second coder to ensure trustworthiness of the coding process and appropriate reflection of all ideas. Code schemes and themes were discussed by the researchers until consensus was reached.

In the last phase code scheme 1 and 3 were compared to determine how quality aspects identified by clinicians corresponded to or differed from the Australian and Dutch standards. Quality aspects mentioned by both, supervisors or trainees and the standards, were categorized as match. Standards lacking quality aspects that were mentioned by supervisors or trainees were categorized as mismatch.

### Confidentiality and ethical considerations

This study was approved by the Dutch NVMO ethical review board on November 10, 2016, record number 798, and by the AMC Ethics Committee on December 9, 2016 using the National Statement on Ethical Conduct in Human Research. Data collection was conducted from December 2016 to November 2017.

## Results

Two evident matches and two mismatches were noticed in the way stakeholders encounter accreditation standards in practice. In addition, analysis of trainee and supervisor perspectives identified 18 important quality aspects for inclusion in standards (Table 2). These aspects were compared with the standards of each country and identified two additional mismatches.

**Table 2:** Overview of the quality aspects mentioned by clinicians

		The Netherlands			Australia					
		TR	CS	ST		TR	CS	ST		
Match	1. Supervision and feedback	X	X	X		X	X	X		
	Quality and effectiveness of supervision and feedback; and accessible and dedicated supervisors were main topics									
	2. Safe learning environment	X	X	X		X	X		X	
	A safe culture and an anti-hierarchical environment were fundamental conditions for training *“Hierarchy is a dangerous system.”* (SupervisorNL02)									
	3. Training program structure	X	X	X		X	X			X
	The training program should be relevant and fit for purpose. It should also be well-integrated with healthcare, continuous professional development and undergraduate education									
	4. Clinical exposure	X	X	X		X	X			X
	Clinical experience in terms of exposure to diverse diagnoses and patient mix were important									
	5. Knowledge and skills	X	X	X		X	X			X
	All clinicians focused on this theme, but trainees especially underlined CanMEDS roles									
	6. Assessment	X		X		X	X			X
	Assessments should be legitimate and fit for purpose, especially if they are summative. The Australian supervisors have the strongest focus on assessment compared to all other groups									
	7. Regulation and governance	X	X	X			X			X
	Supervisors in both countries emphasized the importance of hospital administration understanding and valuing teaching and learning. Without managerial support of a hospital, having a training program would be difficult. In addition, a regulatory framework with an external review of the program is necessary									
	8. Collaboration	X	X	X		X				X
	Australian stakeholders focus on international collaboration, whereas Dutch stakeholders highlight regional collaboration									
	9. Education	X		X		X				X
	Trainees demanded protected time for formal education. A focus on life-long learning, and a variety of learning methods, were important aspects according to accreditors. *“(…) it needs to be able to set up a culture of continual learning. So it is important that once someone has qualified in that specialty that that isn’t the end of their learning as long as their career pathway goes.”* (AccreditorAU03)									
Mismatch	10. Balance between service provision and training	X	X			X	X			
	This is a major issue for clinicians. This balance must be weighted to training according to trainees. Both standards did not cover this aspect									
	11. Trainee empowerment	X				X				
	Trainees plead not to abuse their position and take advantage from their dependency. They need positive supportive behavior from their supervisors. *“(…) generally people that enter into these things are smart and motivated and will do what they need to do to succeed and it’s all about making sure that they're not taken advantage of in that time.”* (TraineeAU01)									
Differences between jurisdictions	12. Resources	X	X	*		X	X			X
	Without proper resources, no proper training was the maxim of the most stakeholders. Dutch standards did cover facilities, but did not capture time and finances, which were the aspects especially underlined by Dutch clinicians									
	13. Job satisfaction	X	X			X	X			*
	Different aspects such as happiness, well-being and good mutual relations were pointed out. Trainee well-being is covered by Australian standards									
	14. Adaptive professionals and training programs		X							X
	Continuous renewal of training programs and professionals adaptive to changing circumstances and future care were essential									
	15. Outcome focused					X	X			X
	In this theme program and graduate outcomes were emphasized. For Dutch accreditors more focus on outcomes was desirable									
	16. Community needs						X			X
	In Australia, alignment to community needs was a key issue in designing and delivering the training program. However, how to approach this meaningfully is a question mark. *“Often trained consumer representation could be too optimistic*.” (AccreditorAU05)									
	17. Access and selection						X			X
	This theme referred to fair selection procedures and access to several training sites									
	18. Trainee-centered program	X	X	X						X
	Training programs could be tailored based on individual talents and competences									

*TR* trainees, *CS* clinical supervisor, *ST* standards

In the following sections, we will elaborate further on the above-mentioned *two matches*: (1) accreditation is necessary (2) trainees are the best source for quality measures, and *four mismatches*: (3) fundamental training aspects that accreditation standards do not capture (this section addresses two mismatches in quality aspects) (4) using standards lack dynamism and (5) quality improvement; driven by standards or intrinsic motivation of healthcare professionals.

### Accreditation is necessary

All participants acknowledged the necessity of accreditation to evaluate quality of training, despite its substantial costs, time-consuming nature, and emotional burden. Many participants argued that without standards it would be difficult to assure a minimum level of quality.

“We have to guarantee a minimum quality for the society. (…) We, humans are fallible.” (AccreditorNL01)

Trainees mentioned the need for an impartial and objective perspective on training quality by an accreditation authority. In addition, supervisors find accreditation reports useful to demand changes or resources from the hospital administration.

“I recognize completely that there is a forcing function there. And I don’t think that is always a bad thing. (…) So, we were able to negotiate some things with hospital administrators that we’ve not been able to achieve traction on, because of the recommendations of the accreditation team (…).” (SupervisorAU04)

The perspectives between Australian and Dutch stakeholders were in contrast when they speak about accreditation as a lever for change. Australian stakeholders believed that accreditation was an enabler and driver of change. The accreditation authority had far more influence on the colleges than faculty members. Without the help of the accreditation authority, colleges were not likely to be able to eliminate differences between programs.

“So many hospitals are acting on historical, you know, eh- a custom. We always did it this way, you know.” (AccreditorAU02)

Dutch faculty members were more skeptical about the influence of accreditation. They were positive about accreditation driving reflection, however they claimed that they did not change their training method or vision after a site visit.

“I told you already, I am not fund of rules/regulations (…) I even do not know the rules (…) do not need to know them (…) I am a passionate supervisor for ten years now.” (SupervisorNL03)

Most supervisors emphasized that severe violation of standards by peers requires penalties, such as withdrawal of accreditation, replacing the program director, and decreasing self-regulation. In contrast, trainees did not consider punishment of non-compliant programs as beneficial, because this could establish a culture of fear and stop professionals from speaking up.

Although different participants believed that standards cannot induce radical change in or capture the culture of the training site, most supervisors and accreditors regarded standards as quite powerful for highlighting important aspects for education providers and training sites. Exclusion of subjects from standards could give the impression that these aspects of training are insignificant.

“And you can’t, you can’t set the standards for culture on a piece of paper. You can’t say, a department must have good culture.” (SupervisorAU04)

### Trainees are the best source of quality measures

Participants considered trainees the best information source regarding training quality. Trainees’ information and feedback were seen as able to negate any window dressing by education providers. Questions were however raised about the conditions for collecting information from trainees, and particularly trainees’ capacity to speak up without assurances of confidentiality and anonymity.

“Keep your mouth, get the approval [successful accreditation].” (AccreditorNL03)

Some supervisors were concerned about trainees giving desirable answers to please accreditation surveyors. Despite the difficulty of the hierarchical environment, trainees were aware of their potential impact and felt responsible to give input.

“(…) it’s pretty hard I think for ehm, for trainees to speak out against stuff [harassment and bullying amongst other] like that, particularly where it’s hard to get into that specialty, and they feel like, you know, whistle-blowers in medicine are treated horrifically. So, ehm, I think, I think getting people to give you straight answers in an interview, that situation would be quite difficult (…).” (TraineeAU01)

### Fundamental training aspects that accreditation standards do not capture

There were two evident mismatches in the quality aspects: service provision and training balance, and trainee empowerment. Neither of both standards covered these aspects, however their inclusion is supported, especially by trainees.

Trainees mention that service provision is often prioritized over training. Although they learn from providing care, learning from clinical work demands allocated time. The appropriate balance between workload and active and reflective learning needs to be secured.

“The training posts that I’ve now found most valuable are often the ones that have an appropriate workload, such the trainees are doing enough cases to gain clinical experience, but also spending some time discussing those cases with their consultants.” (TraineeAU04)

In the context of training aspects, trainees plead to be empowered and supported by their supervisors to speak up. Trainees are in a vulnerable and dependent hierarchical position; others taking advantage of them can impact their well-being, learning or performance. Mentoring may be beneficial for problems trainees experience during their training.

“(…) generally, people that enter into these things [training programs] are smart and motivated and will do what they need to do to succeed and it’s all about making sure that they are not taken advantage of in that time.” (TraineeAU01)

“We have to move towards a system where it is accepted to show vulnerability and a system dependent on trainees. Let them [programs] earn their trainees.” (TraineeNL01)

### Using standards lack dynamism

Supervisors encountered too much rigidity with some accreditation standards. Ideally, the context of practice should be more emphasized. They would prefer more room for flexibility and negotiation, for example collegial peer review, in which clinical supervisors are encouraged to show best practices. Accreditors had similar perspectives; accreditation should be more dynamic to continuously adapt to changing circumstances, and accreditation authorities should not hinder, but support training programs.

“Visiting a program should not be an assessment, in my opinion it should be a collegial process to reflect together on the training program with the framework [standards] which we agreed upon. (…) So, if the visit is intended as a thorough check of my training program, then I’ll say well, that’s unsupportive and a sign of distrust.” (SupervisorNL01)

### Improvement: driven by standards or intrinsic motivation of healthcare professionals

On the topic of quality improvement accreditors and clinicians have contrasting views. Accreditors in both jurisdictions believed firmly in standards being a driving force for quality improvement, although one Dutch accreditor questioned whether a high-stake summative assessment should be the approach.

Clinical supervisors and some trainees, however, mentioned that quality improvement should be driven by healthcare professionals and could not solely be achieved by an external authority. Many supervisors argue that setting standards is inherent to minimum acceptable norms and accreditation authorities should rather not go beyond their scope.

“But again, it’s about enabling the minimum standard and hopefully then ehm, allowing people to find, move beyond that to a higher standard.” (SupervisorAU04)

## Discussion

This study explored the perspectives of trainees, clinical supervisors and accreditors on quality of training, accreditation and standards in Australia and the Netherlands. We explored the differences and similarities in their views and compared this to the accreditation standards of both countries. Two matches and four mismatches were identified. In this part, we will reflect on our findings using the three quality management approaches: quality assurance, quality control and quality improvement.

The participants’ perceived necessity of accreditation is a remarkable acceptance of accreditation considering the evidence-based era. Despite the lack of robust evidence for accreditation effectiveness and impact in the current literature, Brubakk et al. claimed that accreditation is not likely to be abandoned [24]. Efficacy of accreditation may not be linked to evidence; however, the social meaning may shed light on its efficacy. The clinicians’ perceived necessity of accreditation could originate in the legitimacy of the accreditation authority and therefor clinicians could respect its power [25]. It is also plausible that clinicians will comply with most standards even if there were no sanctions or legitimacy, because these standards fit their own ambition [26]. Social identity and motivation to protect the medical profession could be another explanation for requiring accreditation [27]. This may explain the supervisors’ desire for penalties in cases of severe violation of standards.

Participants acceptance of accreditation was regardless of the used quality management approach(es); however, the clinicians and the accreditors did not agree whether or not quality improvement should be driven by accreditation. Ideally, compliance with standards should lead to continuous quality improvement in which training programs strive for excellence [2, 28–30]. Although quality improvement is valued in many jurisdictions, quality improvement is not solely achieved by enforcing standards, since it requires a degree of (intrinsic) motivation of all concerned [31–33]. This was also confirmed by supervisors in this study. Research suggests that whether accredited persons’ intrinsic motivation is enhanced or undermined depends on how the accredited party perceives the accreditation authority’s standards and actions [31, 33]. If standards are perceived as fitting in a shared ambition this may increase the intrinsic motivation of the accredited party [31]. One study among Danish general practitioners (GPs) has shown that accreditation may even foster intrinsic motivation as long as GPs perceive accreditation as a quality improvement instrument [33]. Encouraging (continuous) quality improvement, means continuous (small) modifications and changes on the workplace. Change is challenging, but by merely developing standards and policy-making, without an implementation strategy for the clinical workplace it is hard to initiate willingness for change. This also aligns with leading change model of Kotter: a sense of urgency must be established, the vision must be created and communicated, empowerment to act on vision and improvement must be consolidated [34].

Australian standards covered more aspects than Dutch standards. This could be typical for a relatively new accreditation system which is based upon state of the art and societally responsible accreditation design and not constrained by a lot of historical influences. We believe that there are two other reasons for the differences: (re)current political debate and traditions. Access and selection are not included in the Dutch standards and equity and diversity are less prominent in the political debate than in Australia. Other differences like no standards for job satisfaction, outcome focused programs and community needs in the Netherlands could be explained by traditions, which are difficult to change. Nowadays, if an accreditation system was newly introduced in the Netherlands this would be most probably included in the standards. Trainee-centered programs are aligned with the strong educational influence from the Maastricht School with a large emphasis on student-centered education in the Netherlands [35].

If we compare Table 2 with the comprehensive WFME global standards for medical specialist training, the standards miss the following items: requirement for international or regional collaboration, allocated time for formal education, trainee empowerment, job satisfaction and trainee-centered programs [36]. In the development of accreditation, comparing international standards is an effective way of learning from the best practices of each other.

Most of the current fundamental quality aspects were captured by the standards. However, according to Koksma & Kremer quality is not an objective truth, but dynamic [37]. Quality aspects are subject to changing perspectives, for instance social responsibility is probably an emerging aspect. If we accept that quality is dynamic, the quite rigid accreditation standards must make room for flexible components. Supervisors and accreditors agreed upon flexibility regardless of the used quality management approach. We believe that accreditors may be key figures in adding more dynamics to accreditation standards by bridging between clinical and regulatory practice.

This study uses trainees’ views in measurement of quality and found that trainee feedback and information is the most important tool. This is relevant for setting up a quality control approach  that includes the output of the process. It is also common to search for consumers’ perspectives in evaluation. This study has taken trainees to be active participants in their learning rather than consumers. In the regulation of medical specialist training, public health services and patients’ interests are also at stake. While patients may be seen as primary consumers of outcomes of training, it is not always easy to embed their perspectives in training evaluation. Contrary to the Dutch accreditation processes, the Australian accreditation processes and standards do require education providers to engage health consumers and the community on defining educational purpose and program outcomes as well as in program evaluation. Australian specialist training providers are responding to these standards in multiple ways. The right instruments for patient and consumer feedback could enhance relevance and acceptability of their perspectives for training evaluation [38].

While trainee feedback is seen as a source of valuable information; the Australian and Dutch standards lack trainee empowerment. This could be a call for at least a quality assurance approach on the area of trainee empowerment. It is conceivable that the culture in which the training occurs is hierarchical and less likely to provide opportunities for feedback and criticism. Besides that, trainees are in dependent and vulnerable positions, which make them unwilling to speak up to seniors [39, 40]. By including trainee empowerment in standards, it does not necessarily lead to an immediate change, but at least it may highlight trainee empowerment as an important aspect of training.

### Strengths and limitations

This study contributes to the scarce empirical knowledge about accreditation for medical specialty training. Participants were from different institutions in two countries. It is reasonable to assume that our findings will have some relevance and potential transferability to other settings; nevertheless, the relevance may be questioned in other contexts or cultures. This study is based on a socio-constructivist approach, quantitative proof for accreditation is still limited.

### Suggestions for further research

Although we gained insights in clinicians’ perspectives about quality and accreditation of training, it would be interesting to explore the effect of standards addressing trainee empowerment in the clinical environment. Moreover, in order to improve accreditation systems, further research should focus on the value of flexible components in the accreditation design. For example, reducing the number of specific standards and focusing more on relevant themes, such as teaching and learning methods, or greater co-design of the process between the accreditors and the organization being accredited.

### Practical implications

Our findings raise intriguing questions regarding the nature and extent of the current accreditation design. Future accreditation design should be more aligned with the dynamic concept of quality.

## Conclusion

Accreditation standards change in a less dynamic fashion than the context and perspectives of clinicians, however clinicians did not dispute the utility of accreditation. Flexibility in accreditation standards and processes is needed to keep pace with the developments in and beyond the workplace. Empowering trainees as partners in learning and decision-making about training might add to the quality of training sites and needs to be addressed by the accreditation system. Improvement on trainee empowerment is required, especially since  trainees are considered the most valuable feedback source. Accreditation systems could also benefit from taking account of the internal drivers of clinicians.

## Data Availability

The datasets used and/or analyzed during the current study are available from the corresponding author upon reasonable request. The data are partially in Dutch and partially in English.
